# TGF*β*1-Induced Differentiation of Human Bone Marrow-Derived MSCs Is Mediated by Changes to the Actin Cytoskeleton

**DOI:** 10.1155/2018/6913594

**Published:** 2018-02-15

**Authors:** Mona Elsafadi, Muthurangan Manikandan, Sami Almalki, Mohammad Mobarak, Muhammad Atteya, Zafar Iqbal, Jamil Amjad Hashmi, Sameerah Shaheen, Nehad Alajez, Musaad Alfayez, Moustapha Kassem, Raed Abu Dawud, Amer Mahmood

**Affiliations:** ^1^Stem Cell Unit, Department of Anatomy, College of Medicine, King Saud University, Riyadh, Saudi Arabia; ^2^KMEB, Department of Endocrinology, University Hospital of Odense, Odense, Denmark; ^3^KMEB, Department of Endocrinology, University of Southern Denmark, Odense, Denmark; ^4^College of Agriculture, King Saud University, Riyadh, Saudi Arabia; ^5^Department of Histopathology, College of Medicine, King Saud University, Riyadh, Saudi Arabia; ^6^Department of Histology, Faculty of Medicine, Cairo University, Cairo, Egypt; ^7^Department of Basic Sciences, College of Applied Medical Sciences, King Saud Bin Abdulaziz University for Health Sciences (KSAU-HS), National Guard Health Affairs, Al Ahsa, Saudi Arabia; ^8^Center for Genetics and Inherited Diseases, Taibah University, Medina, Al Madinah, Saudi Arabia; ^9^Department of Comparative Medicine, King Faisal Specialist Hospital and Research Centre, Riyadh, Saudi Arabia

## Abstract

TGF*β* is a potent regulator of several biological functions in many cell types, but its role in the differentiation of human bone marrow-derived skeletal stem cells (hMSCs) is currently poorly understood. In the present study, we demonstrate that a single dose of TGF*β*1 prior to induction of osteogenic or adipogenic differentiation results in increased mineralized matrix or increased numbers of lipid-filled mature adipocytes, respectively. To identify the mechanisms underlying this TGF*β*-mediated enhancement of lineage commitment, we compared the gene expression profiles of TGF*β*1-treated hMSC cultures using DNA microarrays. In total, 1932 genes were upregulated, and 1298 genes were downregulated. Bioinformatics analysis revealed that TGF*β*l treatment was associated with an enrichment of genes in the skeletal and extracellular matrix categories and the regulation of the actin cytoskeleton. To investigate further, we examined the actin cytoskeleton following treatment with TGF*β*1 and/or cytochalasin D. Interestingly, cytochalasin D treatment of hMSCs enhanced adipogenic differentiation but inhibited osteogenic differentiation. Global gene expression profiling revealed a significant enrichment of pathways related to osteogenesis and adipogenesis and of genes regulated by both TGF*β*1 and cytochalasin D. Our study demonstrates that TGF*β*1 enhances hMSC commitment to either the osteogenic or adipogenic lineages by reorganizing the actin cytoskeleton.

## 1. Introduction

Fat and bone tissues both originate from bone marrow progenitor cells called skeletal stem cells, also known as bone marrow-derived multipotent stromal cells or mesenchymal stem cells (MSCs). The formation of these tissues is regulated throughout an organism's lifetime by homeostatic mechanisms within the marrow cavity. It has been suggested that an imbalance between osteogenic and adipogenic lineage commitment and differentiation is responsible for age-related impairment of bone formation, and a number of therapeutic interventions targeting and activating MSCs, thus enhancing bone mass, have been proposed. Indeed, the identification of novel strategies to steer human skeletal (mesenchymal) stem cell differentiation towards the production of osteoblastic cells, thus increasing bone formation, is very topical in the bone biology field.

The transforming growth factor (TGF) superfamily consists of over 40 members, including activins, inhibins, bone morphogenetic proteins (BMPs), growth and differentiation factors (GDFs), and TGF*β*s [1]. TGF family members are multifunctional regulators of cell growth and differentiation, playing pivotal roles during embryonic development, organogenesis, and tissue homeostasis [2]. The cytokine TGF*β*1 is among the most abundant in bone matrix [3] and is secreted by endothelial cells, epithelial cells, fibroblasts, smooth muscle cells, and most immune cells [4]. TGF*β*1 is deposited in bone matrix as an inactive, latent complex with latency-associated protein (LAP), the binding of which masks the receptor domains of active TGF*β*1. During bone formation, osteoclast-mediated bone resorption activates TGF*β*1 by cleaving LAP and releasing it from bone matrix, thus creating a transient gradient of active TGF*β*1 that attracts MSCs to bone remodeling sites, where they undergo osteoblastic differentiation [5]. Furthermore, TGF*β*1 is known to regulate the proliferation and differentiation of osteoprogenitor cells [6].

Actin microfilaments are composed of polymers of actin, the most abundant cellular protein which also forms the thinnest part of the cytoskeleton, and are primarily responsible for skeletal structure [7]. Cellular actin exists in two forms, filamentous polymerized actin (F-actin) and globular/monomer depolymerized actin (G-actin), and transitions between these forms during highly dynamic intracellular polymerization and depolymerization processes [8]. In mammals, actin polymerization factors regulate actin polymerization and depolymerization [9]. While the stiffness of actin is lower than that of microtubules, actin molecules form a highly organized structural network, supported by a large number of interacting cross-linking proteins, which together confer a substantial amount of mechanical strength [10]. The cytoskeleton is known to be important for determining cell morphology and for mediating changes in adhesion and differentiation [11]. Indeed, during human MSC (hMSC) lineage commitment, cells undergo significant morphological changes and actin cytoskeletal reorganization which contribute to the determination of cellular fate [7, 12].

In this study, we investigated the effect of TGF*β*-induced actin cytoskeleton modifications on the potential of hMSCs to differentiate into osteogenic and adipogenic lineages, as well as the effect of the actin polymerization inhibitor cytochalasin D (CYD). Our data suggest that TGF*β*-induced actin cytoskeleton reorganization is a prerequisite for hMSC differentiation into osteocytic or adipocytic lineages.

## 2. Results

### 2.1. TGF*β*1 Treatment Enhanced the Osteogenic Differentiation of hMSCs

**Figure 1:**
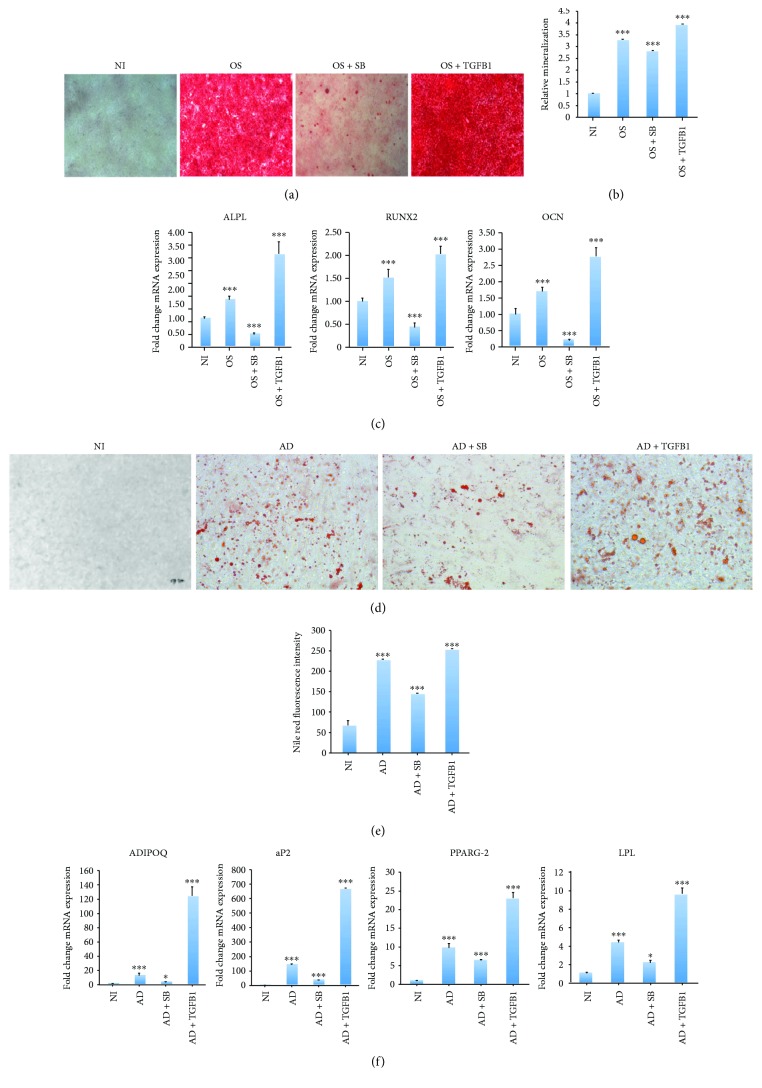
TGF*β*1 induces osteogenic and adipogenic differentiation. MSCs underwent osteogenic or adipogenic differentiation by culturing cells in the appropriate medium for 7 days. (a) Micrographs showing the degree of mineralized calcium deposition in noninduced cells (NI), osteoinduced cells (OS), osteoinduced cells + SB-431542 (OS + SB), and osteoinduced cells + TGF*β*1 (OS + TGFB), as assessed by alizarin red S staining (20x magnification). (b) Quantification of mineralization in the alizarin red S stained groups shown in (a). Data are shown as the mean ± SD of three independent experiments (^∗∗∗^ *p* < 0.005). (c) mRNA expression of the osteogenic markers *ALPL*, *RUNX2*, and *OCN*, normalized to GAPDH, as determined by RT-PCR. Data are shown as the mean ± SD of three independent experiments (^∗^ *p* < 0.05; ^∗∗∗^ *p* < 0.0005). (d) Micrographs showing the accumulation of lipid droplets in noninduced cells (NI), adipoinduced cells (AD), adipoinduced cells + SB-431542 (AD + SB), and adipoinduced cells + TGF*β*1 (AD + TGFB), as determined by Oil red O staining (20x magnification). (e) Quantification of mature adipocytes in the NI, AD, AD + SB, and AD + TGFB groups, as determined by Nile red fluorescence intensity. Data are shown as the mean ± SD of three independent experiments (^∗∗∗^ *p* < 0.005). (f) mRNA expression of the adipogenic markers *LPL*, *aP2*, *PPARG-2*, and *ADIPOQ*, normalized to GAPDH, as determined by RT-PCR. Data are shown as the mean ± SD of three independent experiments (^∗^ *p* < 0.05; ^∗∗∗^ *p* < 0.0005).

A single treatment with TGF*β*1 (10 ng/ml, for 2 days) enhanced hMSC osteogenic differentiation, as shown by the increased mineralized matrix formation made evident by alizarin red S staining (Figures 1(a) and 1(b)). Conversely, when TGF*β*1 signaling was blocked with the inhibitor SB-431542 (10 *μ*M), significantly lower mineralized matrix formation was observed (Figures 1(a) and 1(b)). Consistent with this, higher expression of the osteoblastic genes alkaline phosphatase (*ALP*), runt-related transcription factor 2 (*RUNX2*), and osteocalcin (*OCN*) was observed in hMSCs undergoing osteogenic differentiation in the presence of TGF*β*1, while treatment with the TGF*β*1 inhibitor SB-431542 severely inhibited this expression (Figure 1(c)).

### 2.2. TGF*β*1 Treatment Enhanced the Adipogenic Differentiation of hMSCs

Next, we examined the effect of treating hMSCs with a single dose of TGF*β*1 (10 ng/ml, for 2 days) on adipogenic differentiation. We found that adipogenic differentiation was enhanced following TGF*β*1 treatment, as shown by an increase in the number of lipid-filled adipocytes (Figures 1(d) and 1(e)). Similarly, the expression of several adipogenic gene markers, including lipoprotein lipase (*LPL*), peroxisome proliferator-activated receptor gamma 2 (*PPARG-2*), adipocyte protein 2 (*aP2*), and *ADIPOQ*, was upregulated following TGF*β*1 treatment, while treatment with SB-431542 reversed these effects (Figure 1(f)).

### 2.3. TGF*β*1 Stimulation Has No Effect on hMSC Viability or Proliferation

**Figure 2:**
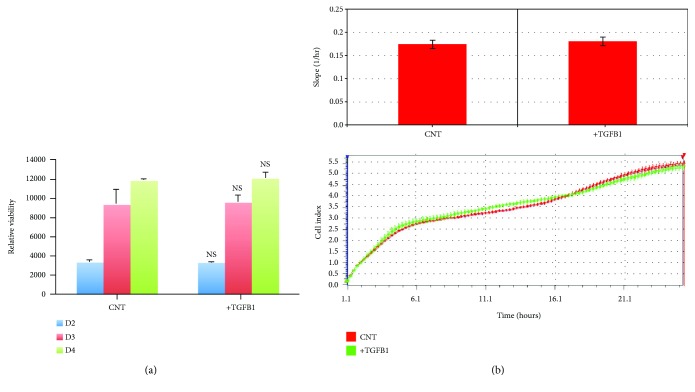
TGF*β*1 does not affect hMSC proliferation or viability. (a) Chart showing the relative hMSC viability in the absence (CNT) or presence (TGFB1) of TGF*β*1, as determined by alamarBlue assay reagent. Shown are cell viabilities on days 2 (D2), 3 (D3), and 4 (D4) of culture. (b) Real-time proliferation assay data using the xCELLigence RTCA DP system for hMSC cells with and without TGF*β*1 treatment. Lower panel: cell proliferation was measured at 15-minute intervals for a total duration of 24 hours. Upper panel: summary data showing cellular proliferation after 24 hours. Data are shown as the mean ± SD of two independent experiments (*n* = 6). NS: not significant.

The effect of TGF*β*1 on hMSC cell viability was assessed using alamarBlue assay reagent. No significant effect on viability was observed after 4 days of treatment (Figure 2(a)). To investigate the effect of TGF*β*1 on cellular proliferation, we used the xCELLigence RTCA DP® cell proliferation assay system, which allows the continuous monitoring of cell numbers over time. As shown in Figure 2(b), there was no measurable difference in hMSC proliferation in the presence or absence of TGF*β*1.

### 2.4. Molecular Phenotype of TGF*β*1-Treated hMSCs

**Figure 3:**
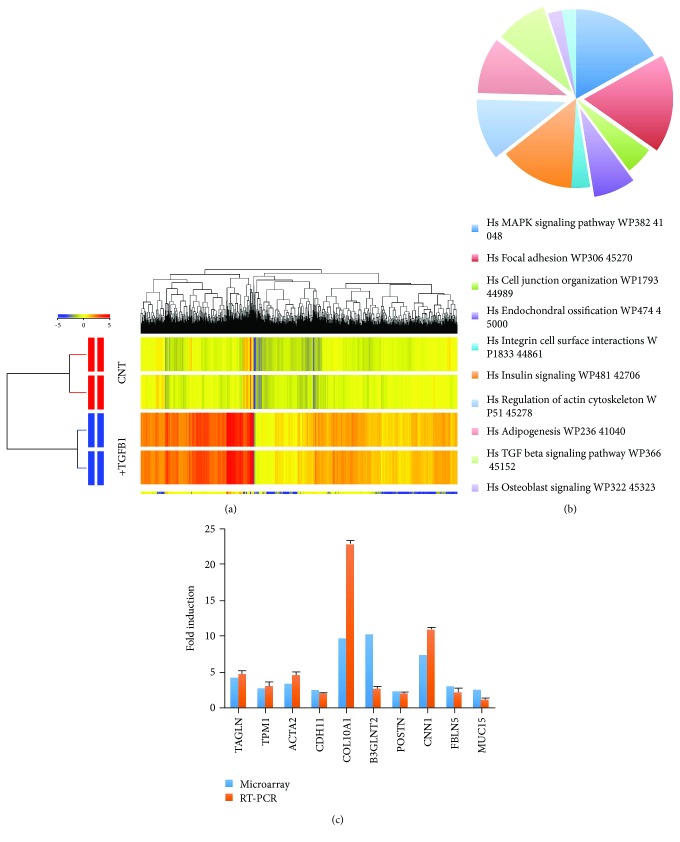
Molecular phenotype of TGF*β*1-treated hMSCs. (a) Hierarchical clustering of genes that were differentially expressed in TGF*β*l-treated and untreated control (CNT) hMSCs. Rows represent individual gene expression for duplicate treated and untreated samples, as indicated. Columns represent individual transcripts. Relative expression levels are presented colorimetrically, according to the scale shown in the color bar. (b) Pie chart showing the pathways with the highest enrichment of genes significantly upregulated in TGF*β*1-treated cells. (c) qRT-PCR validation of selected genes that were upregulated in the microarray data (*n* = 3, ^∗^ *p* < 005; ^∗∗∗^ *p* < 0001). Cells treated with vehicle (DMSO) were used as controls.

To understand the molecular mechanisms underlying the TGF*β*l-mediated regulation of hMSC differentiation, we compared global gene expression in TGF*β*l-treated hMSCs and vehicle-treated control cells using microarray analysis. In total, 1932 gene transcripts were significantly upregulated, and 1298 were significantly downregulated following TGF*β*l treatment. Significant changes were defined as a fold change ≥ 2, *p* < 0.05 and are listed in Supplementary Tables S1 and S2. Hierarchical clustering of differentially expressed genes revealed a clear distinction between TGF*β*l-treated and control samples (Figure 3(a)). Next, we used performed gene ontology analysis to identify the biological processes that were favored following TGF*β*l treatment. We found that the genes that were significantly altered in TGF*β*l-treated MSCs were enriched within several skeletal and extracellular matrix categories, including extracellular matrix (53 genes), extracellular matrix organization (51 genes), and proteinaceous extracellular matrix (Supplementary Table S3). Furthermore, pathway analysis of significantly changed genes revealed the significant enrichment of several signaling pathways in TGF*β*l-treated hMSCs. Among these, the most enriched pathways were “regulation of actin cytoskeleton,” “MAPK signaling,” “focal adhesion,” “TGF*β*1 signaling,” “adipogenesis,” “endochondral ossification,” and “osteoblast signaling” (Figure 3(b)).  Table 1 lists the genes within osteogenesis- and adipogenesis-related signaling pathways that were upregulated in TGF*β*1-treated cells. A selected panel of genes known to be involved in cell differentiation and TGF*β* signaling that were significantly changed in the microarray data were examined by qRT-PCR. In general, a good degree of concordance was observed between the microarray and qRT-PCR data (Figure 3(c)).

### 2.5. Actin Microfilaments in MSCs Are Altered following Treatment with TGF*β*1 or the Actin Polymerization Inhibitor CYD

**Figure 4:**
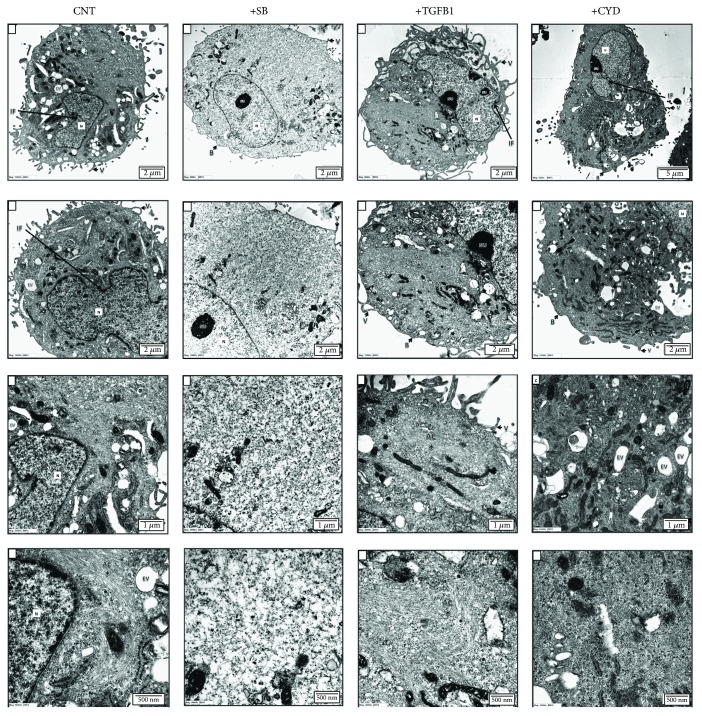
Transmission electron microscopy of MSCs with and without treatment with SB-431542, TGF*β*1, or CYD. TEM ultrastructural analysis of MSCs following no treatment (CNT) or treatment with SB-431542 (SB), TGF*β*1, or CYD. Increasing levels of magnification are indicated by scale bars. N: nucleus; Nu: nucleolus; AC: actin filaments; V: microvilli; M: mitochondria; PL: primary lysosome; SL: secondary lysosome; rER: rough endoplasmic reticulum; G: Golgi bodies; B: cell blebs; P: cell processes; IF: nuclear membrane infolding; EV: endocytotic vacuole.

Our molecular phenotyping analysis of TGF*β*l-treated hMSCs revealed a significant enrichment of genes associated with cytoskeletal changes. Based on this, and on our previous observations that TGF*β*l treatment triggers significant morphological changes in hMSCs, we examined the effect of TGF*β*l on the cytoskeleton using transmission electron microscopy (TEM), which has the power to reveal structural changes in actin microfilaments. Actin microfilament polymerization was found to be inhibited in cells treated with either the potent actin polymerization inhibitor CYD or the TGF*β* inhibitor SB-431542. In contrast, TGF*β*1 treatment was associated with a prominent distribution of actin filaments, organized as bundles/aggregates, in the perinuclear area and at one cell pole (Figure 4). The ultrastructural characteristics of the cells under the various treatment conditions are summarized in Supplementary Table S4.

### 2.6. CYD Regulates Osteogenic and Adipogenic Differentiation in the Presence of TGF*β*1

**Figure 5:**
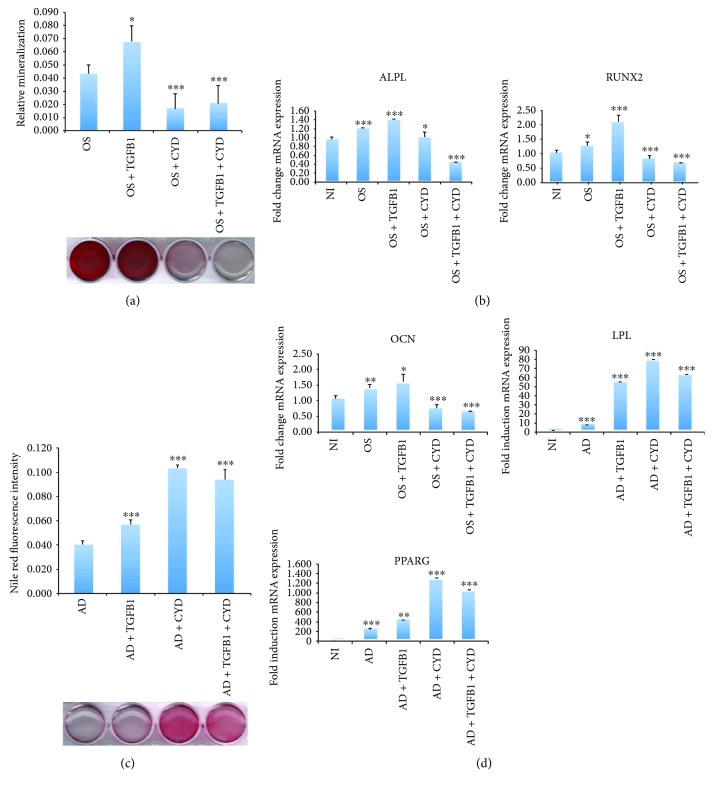
Inhibition of actin polymerization promotes adipogenic differentiation but inhibits osteogenic differentiation in MSCs. MSCs underwent osteogenic or adipogenic differentiation by culturing cells in the appropriate medium for 7 days. Cells also underwent the indicated treatments. (a) Mineralized calcium deposition, as determined by alizarin red S staining in MSCs that were osteoinduced (OS), osteoinduced with TGF*β*1 treatment two days prior to induction (OS + TGF*β*1), osteoinduced with CYD treatment at the onset of induction (OS + CYD), or osteoinduced with both TGF*β*1 and CYD treatment at the time points described above (OS + TGF*β*1 + CYD). Lower panel: micrograph of stained wells. Upper panel: quantification of mineralized matrix formation under the indicated treatment conditions. Data are shown as the mean ± SD of three independent experiments (^∗^ *p* < 0.05; ^∗∗∗^ *p* < 0.005). (b) Gene expression of the osteogenic markers *ALPL*, *RUNX2*, and *OCN*, normalized to GAPDH, as determined by qRT-PCR. Cells were either not induced (NI) or induced under the conditions described in (a). Data are shown as the mean ± SD of three independent experiments (^∗^ *p* < 0.05; ^∗∗^ *p* < 0.005, ^∗∗∗^ *p* < 0.0005). (c) Adipogenic differentiation of MSCs that were adipoinduced (AD), adipoinduced with TGF*β*1 treatment 2 days prior to induction (AD + TGF*β*1), adipoinduced with CYD treatment, initiated at the onset of induction (AD + CYD), or adipoinduced with both TGF*β*1 and CYD treatment at the time points described above (AD + TGF*β*1 + CYD). Lower panel: Oil red O staining of the indicated cells. Upper panel: Nile red quantification of oil content under the indicated conditions. (d) Gene expression of the adipogenic marker genes *PPARG* and *LPL*, determined by qRT-PCR and normalized to GAPDH, under the indicated treatment regimens. Data are shown as the mean ± SD of three independent experiments (^∗∗^ *p* < 0.005, ^∗∗∗^ *p* < 0.0005). All controls were treated with vehicle only.

To confirm that TGF*β*1 regulates actin cytoskeletal dynamics, hMSCs undergoing either osteogenic or adipogenic differentiation were treated with TGF*β*1 in the absence or presence of the actin polymerization inhibitor CYD. CYD treatment significantly inhibited hMSC osteogenic differentiation in both the presence and absence of TGF*β*l, as shown by reduced mineralization (Figure 5(a)). Similarly, expression of the osteogenic marker genes *ALPL*, *RUNX2*, and *OCN* was inhibited by CYD treatment, with and without TGF*β*l (Figure 5(b)). Conversely, CYD treatment enhanced hMSC adipogenic differentiation, as shown by a greatly increased number of lipid-filled mature adipocytes and the increased expression of the adipogenic marker genes *LPL* and *PPARG-2.* These effects were maintained when cells were treated concomitantly with TGF*β*l (Figures 5(c) and 5(d)).

### 2.7. Molecular Phenotype of CYD-Treated Cells

**Figure 6:**
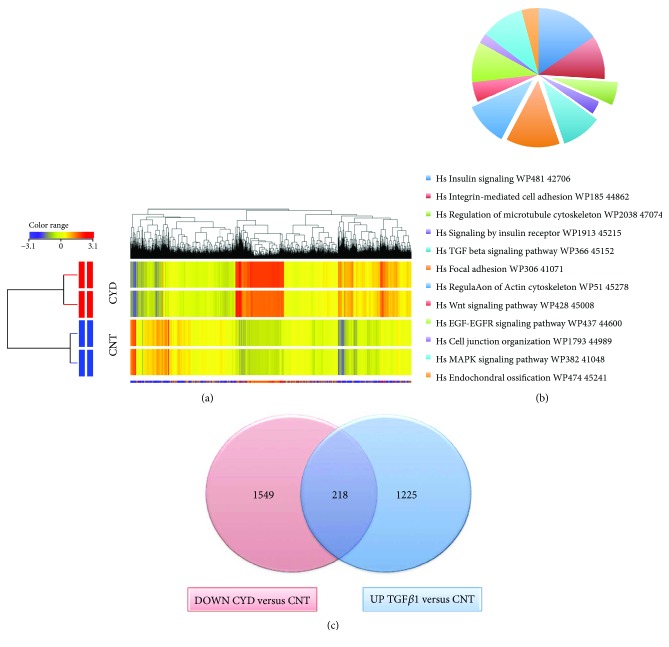
Molecular phenotype of CYD-treated hMSCs. (a) Hierarchical clustering of genes that were differentially expressed in CYD-treated and untreated control (CNT) hMSCs. Rows represent individual gene expression for duplicate treated and untreated samples, as indicated. Columns represent individual transcripts. Relative expression levels are presented colorimetrically, according to the scale shown in the color bar. (b) Pie chart showing the pathways with the highest enrichment of genes significantly upregulated in CYD-treated cells. (c) Venn diagram depicting the overlap between the upregulated genes in TGF*β*1-treated cells (UP TGF*β*1 versus CNT) and the downregulated genes in CYD-treated cells (DOWN CYD versus CNT).

The data presented above suggest that CYD and TGF*β*1 target similar molecular pathways during hMSC osteogenic and adipogenic differentiation. In order to investigate this further and to elucidate the molecular mechanisms underlying the CYD-mediated effects on hMSC differentiation, microarray analysis was performed to establish global gene expression profiles for CYD-treated and controls cells. In total, 10,855 genes were significantly upregulated, and 2523 genes were significantly downregulated following CYD treatment. Genes were defined as significantly changed if they had a fold change ≥ 2 and *p* < 0.05 and are listed in Supplementary Tables S5 and S6. As was seen with TGF*β*1 treatment, hierarchical clustering of the differentially expressed genes revealed a clear distinction between untreated and CYD-treated hMSCs (Figure 6(a)). Pathway analysis of these genes revealed several molecular pathways that were enriched upon CYD treatment (Figure 6(b)). Among the most significant were pathways involved in the regulation of the actin cytoskeleton, focal adhesion signaling, endochondral ossification, TGF*β*1 signaling, regulation of the microtubule cytoskeleton, and MAPK signaling (Figure 6(b)). The genes that are associated with these pathways that were upregulated in CYD-treated cells are listed in Table 2. Forty-two genes that are involved in adipogenesis-related pathways were significantly enriched in CYD-treated cells (Table 3). Interestingly, 218 genes were both upregulated in TGF*β*1-treated hMSCs and downregulated in CYD-treated hMSCs (Figure 6(c)), showing that the molecular signature on CYD treatment is the inverse of that seen with TGF*β*1 treatment and suggesting that these genes may be involved in TGF*β*-mediated cytoskeletal reorganization (Table 4).

## 3. Discussion

TGF*β* is a potent regulator of various biological functions in many cell types, but its effects on hMSC differentiation are, to date, poorly understood. In the present study, we contribute to this understanding and demonstrate that TGF*β* can enhance both osteoblastic and adipocytic lineage commitment by modulating changes to the actin cytoskeleton.

TGF*β*1 is known to regulate the proliferation and differentiation of osteoprogenitor cells [6, 13–15], and it reportedly stimulates bone matrix apposition and bone cell replication [16]. Several studies have demonstrated that TGF*β*1 promotes bone formation *in vitro* by recruiting osteoblast progenitors and inducing bone matrix formation at early stages of differentiation. In addition to this direct regulation of bone formation, TGF*β*1, along with BMPs, enhances *RUNX2* expression at early differentiation stages [17]. This is consistent with our finding that TGF*β*1 promoted osteogenesis and was associated with the upregulation of the osteogenic genes *ALPL*, *RUNX2*, and *OCN*.

Furthermore, we showed that TGF-*β*1 treatment enhanced the *in vitro* adipocytic differentiation of hMSCs. This is consistent with several previously reported studies which demonstrate that TGF*β*1 has a positive effect on adipogenic differentiation under specific culture conditions [18, 19]; an early study considering rat brown adipocytes showed an upregulation of lipogenic enzymes following TGF*β*1 treatment [19].

Our results showed that TGF*β*1 treatment did not affect MSC cell growth *in vitro*. Previously, conflicting results have been published; some studies reported that TGF*β*1 regulated osteoprogenitor proliferation *in vitro* [13, 20], whereas Yu et al. reported that TGF*β*1 treatment strongly inhibited the proliferation of human lung epithelial cells [21]. The mitogenic effects of TGF*β* on cells are reportedly variable; while progressive mitogenesis was stimulated in confluent cells following treatment with 0.15–15 ng/ml TGF*β*, in sparse cultures 0.15 ng/ml TGF*β* exhibited inhibitory effects. However, at all cell densities, 15 ng/ml TGF*β* stimulated collagen synthesis, with this effect being most pronounced when DNA synthesis was declining [22]. Most of the published data on TGF*β* has shown a mitogenic effect on osteoprogenitors [16, 23–26], but relatively few studies have examined the growth inhibitory effect of this cytokine on osteoblast-like cells [27, 28]. It is likely that these contradictory observations reflect the fact that the effect TGF*β* has on cellular proliferation is dependent upon TGF*β* concentration, culture conditions including cell density, the cell model system (tumorigenic versus nontumorigenic), the differentiation stage of the target cell population, and/or the presence of other growth factors.

The cytoskeleton is known to be important for cell morphology and for mediating changes in adhesion and differentiation [11]. Furthermore, significant changes in cytoskeletal components reportedly occur during hMSC lineage commitment and differentiation [7, 11]. While changes in cell shape can be influenced by differentiation, several studies have shown that the differentiation of precommitted mesenchymal stem cells is itself influenced by changes in cellular morphology resulting from the altered expression of cadherins, integrins, and cytoskeletal proteins [29]. Recently, the inhibition of actin depolymerization was shown to enhance both hMSC differentiation into osteoblasts and *in vivo* bone formation, with these effects being mediated by several signaling pathways and involving focal adhesion kinase (FAK), p38, and JNK activation [7]. Furthermore, a separate study reported that the suppression of actin polymerization, a very early event in hMSC differentiation, following the downregulation of p38 MAPK activity, inhibited osteogenesis [30]. Additionally, *α*-smooth muscle actin is important for both the identification of osteoprogenitors in hMSCs and their differentiation fate [31], and Rho GTPase-mediated cytoskeletal modification is essential for controlling hMSC differentiation and migration [32].

On the other hand, adipocytic differentiation is associated with the morphological change from fibroblast-like cells to spherical cells filled with fat droplets [33]. These morphological alterations are also associated with cytoskeletal changes and actin reorganization, which takes place in the early lineage commitment stage, prior to the upregulation of many adipocytic-specific gene markers [34]. The differentiation of hMSCs into the adipocytic lineage *in vitro* is known to be influenced by the cytoskeletal tension that results following actin reorganization [32]. Furthermore, TGF*β*1 Ca^2+^ signaling is known to regulate osteoblast adhesion through enhanced *α*5 integrin expression, the formation of focal contacts, and the mediation of cytoskeleton reorganization [35, 36]. Additionally, the TGF*β*1-mediated stimulation of DNA synthesis in mouse osteoblastic cells is reportedly associated with morphological changes and is accompanied by the enhanced synthesis and polymerization of cytoskeletal proteins [37]. Consistent with this, our data suggests that TGF*β*1 enhances hMSC lineage commitment by regulating the morphology of the actin cytoskeleton, focal adhesion, and endochondral ossification, via the TGF*β*1 and MAPK signaling pathways.

Also consistent with our results are reports that CYD-mediated reductions in actin polymerization stimulate adipogenesis, but inhibit osteogenesis [30], suggesting that cytoskeletal modification is a prerequisite for cell fate determination. Our gene expression profiling revealed that the genes *FGF1*, *FGF2*, and *KRAS*, which commonly regulate actin cytoskeleton reorganization, were upregulated and downregulated in TGF*β*1- and CYD-treated cells, respectively, suggesting that they are involved in the actin polymerization-mediated differentiation of MSCs.

We showed that during osteogenesis, TGF*β*1 treatment reorganized the cytoskeleton, but this reorganization, and thus osteogenesis, could be disturbed by CYD treatment. Conversely, treatment with either TGF*β*1 or CYD promoted adipogenesis. This observation can potentially be explained by considering that TGF*β*1 and CYD promote the formation of different cytoskeleton patterns, both of which support adipogenesis. Alternatively, it is possible that cytoskeletal reorganization leading to adipogenesis can be promoted by both TGF*β*1-dependent and -independent mechanisms, and that CYD-mediated cytoskeletal reorganization cannot override the TGF*β*1-independent mechanism.

**Figure 7:**
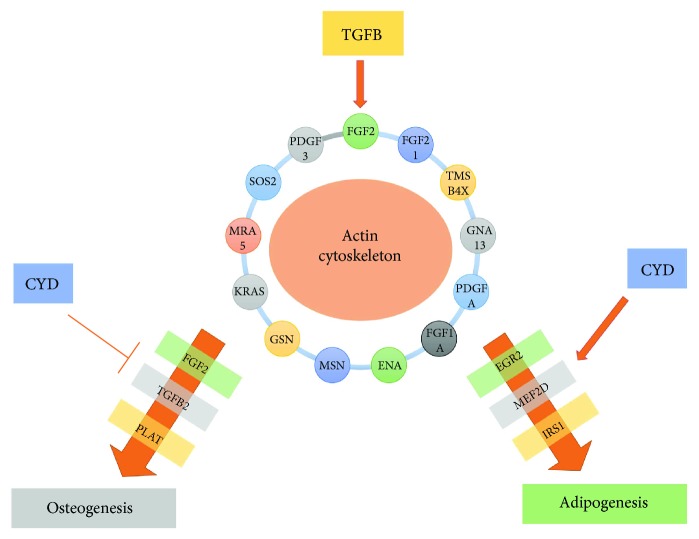
TGF*β*1 signaling in hMSC differentiation. Schematic showing how TGF*β* and CYD affect hMSC osteogenic and adipogenic differentiation through the modulation of genes associated with the actin cytoskeletal pathway. Suggested downstream targets are also shown.

We propose a model wherein TGF*β*1 regulates cytoskeletal organization by modulating actin cytoskeleton-related genes, leading to enhanced hMSC differentiation into both osteoblasts and adipocytes (Figure 7). We propose that CYD enhances adipogenesis and inhibits osteogenesis by regulating the expression of a number of key candidate genes, including *FGF2*, *TGFβ2*, *Plat*, *EGR2*, *MEF2D*, and *IRS1*. These genes were modulated by both TGF*β*1 and CYD and are thus heavily implicated in the determination of hMSC fate. In summary, our study provides novel molecular insights into the role of the intracellular TGF*β* signaling pathway in bone and bone marrow adipose tissue formation. This signaling involves the reorganization of the actin cytoskeleton in order to control the lineage-specific differentiation of hMSCs.

## 4. Materials and Methods

### 4.1. Cell Culture

An hMSC-TERT cell line was created previously to serve as a model of human primary MSCs by overexpressing human telomerase reverse transcriptase (hTERT) in normal human bone marrow MSCs [38]. This cell line has been extensively characterized and exhibits a similar cellular and molecular phenotype to primary MSCs [39]. For the current experiments, we used a previously characterized subline derived from hMSC-TERT cells, termed hMSC-TERT-CL1 [40]. For ease, this cell line is referred to as “hMSC” for the remainder of the manuscript. Cells were cultured in Dulbecco's modified Eagle's medium (DMEM) supplemented with 4500 mg/l D-glucose, 4 mM L-glutamine, 110 mg/l sodium pyruvate, 10% fetal bovine serum (FBS), 1× penicillin/streptomycin (pen/strep), and nonessential amino acids. All reagents were purchased from Gibco, USA.

### 4.2. *In Vitro* Osteoblastic Differentiation

To induce osteoblastic differentiation, cells were initially grown in standard DMEM growth medium in 6-well plates at a density of 0.3 × 10^6^ cells/ml. Once 70–80% confluence was reached, the medium was replaced with DMEM supplemented with osteoblast induction mixture, containing 10% FBS, 1% pen/strep, 50 *μ*g/ml L-ascorbic acid (Wako Chemicals, Neuss, Germany), 10 mM *β*-glycerophosphate (Sigma), 10 nM calcitriol (1*α*,25-dihydroxyvitamin D_3_; Sigma), and 10 nM dexamethasone (Sigma). The medium was replaced 3 times per week. Cells were cultured in standard culture medium in parallel as controls.

### 4.3. *In Vitro* Adipocytic Differentiation

To induce adipocytic differentiation, cells were initially grown in standard DMEM growth medium in 6-well plates at a density of 0.3 × 10^6^ cells/ml. Once 90–100% confluence was reached, the medium was replaced with DMEM supplemented with adipogenic induction mixture, containing 10% FBS, 10% horse serum (Sigma), 1% pen/strep, 100 nM dexamethasone, 0.45 mM isobutyl methylxanthine [41] (Sigma), 3 *μ*g/ml insulin (Sigma), and 1 *μ*M rosiglitazone [42] (Novo Nordisk, Bagsvaerd, Denmark). The medium was replaced 3 times per week, and cells cultured in parallel in standard culture medium were used as controls.

### 4.4. Cytochemical Staining

#### 4.4.1. Alizarin Red S Staining for Mineralized Matrix

Once cells had grown sufficiently, the cell monolayer was washed with phosphate-buffered saline (PBS) and then fixed with 4% paraformaldehyde for 15 minutes at room temperature. Cells were then rinsed 3 times in distilled water and stained with 2% alizarin red S Stain (Cat. number 0223; ScienCell, Carlsbad, CA, USA) for 20–30 minutes at room temperature. Cells were washed further 3–5 times with water to remove excess dye and then stored in water to prevent them drying out. Cells were then visualized using an inverted microscope (ZEISS AX10). To quantify alizarin red S staining, and hence mineralization, plates were air dried, and then the alizarin red S dye was eluted by adding 800 *μ*l acetic acid to each well and incubating for 30 minutes at room temperature, as described previously [43]. The stain was then quantified by measuring the absorbance at 405 nm with an Epoch spectrophotometer (BioTek Inc., Winooski, VT, USA).

#### 4.4.2. OsteoImage Mineralization Assay

The formation of mineralized matrix *in vitro* was quantified using an OsteoImage mineralization assay kit according to manufacturer's instructions (Cat. number PA-1503; Lonza, USA). Briefly, culture medium was removed, and cells were washed once with PBS and then fixed with 70% cold ethanol for 20 minutes. Next, diluted staining reagent was added at a level recommended by the manufacturer, and plates were incubated in the dark for 30 minutes at room temperature. The cells were then washed, and staining was quantified using a fluorescence plate reader (Molecular Devices Co., Sunnyvale, CA, USA) with excitation and emission wavelengths of 492 and 520 nm, respectively.

#### 4.4.3. Oil Red O Staining for Lipid Droplets

Cytoplasmic lipid droplets within mature adipocytes were visualized using Oil red O staining. Cells were washed with PBS, fixed in 4% formaldehyde for 10 minutes at room temperature, rinsed once with 3% isopropanol, and then stained for 1 hour at room temperature with filtered Oil red O staining solution (prepared by dissolving 0.5 g Oil red O powder in 60% isopropanol). In order to quantify mature adipocytes, Oil red O stain was eluted from cells by adding 100% isopropanol to each well, and then the absorbance at 510 nm was measured using an Epoch spectrophotometer.

#### 4.4.4. Nile Red Staining for the Quantification of Mature Adipocytes

A 1 mg/ml stock solution of Nile red fluorescent stain was prepared in DMSO and stored in the dark at −20°C. Cultured undifferentiated and differentiated cells were fixed in 4% paraformaldehyde (Sigma) for 15 minutes and then washed once with PBS. PBS was then removed, and cells were stained with 5 *μ*g/ml Nile red stain in PBS for 10 minutes at room temperature. The fluorescence signal was measured using a SpectraMax/M5 fluorescence spectrophotometer (Molecular Devices Co.) in bottom-well scan mode. Nine readings were taken per well using excitation and emission wavelengths of 485 nm and 572 nm, respectively.

### 4.5. Quantitative Real-Time PCR (qRT-PCR)

Total RNA was extracted from cells using a PureLink RNA mini kit (Cat number 12183018A; Ambion, USA) according to manufacturer's recommendations and quantified using a NanoDrop 2000 spectrophotometer (Thermo Scientific, USA). Complementary DNA (cDNA) was synthesized from 1 *μ*g RNA using a high-capacity cDNA reverse transcription kit (Applied Biosystems, USA) and a MultiGene thermal cycler (Labnet) according to manufacturer's instructions. Relative mRNA levels were inferred from the cDNAs using Power SYBR Green master mix (Applied Biosystems, UK) and TaqMan Universal master mix II, no UNG (Applied Biosystems, USA), both according to manufacturer's instructions, and a real-time PCR detection system (Applied Biosystems). Relative mRNA levels were normalized to the reference gene GAPDH, and then gene expression quantification was performed using a comparative Ct method, wherein *Δ*CT is defined as the difference between the target and reference gene CT values. Primers are listed in Supplementary Tables S7 and S8. These primers were either TaqMan primers (Applied Biosystems) or custom primers whose sequences have been published previously.

### 4.6. Global Gene Expression Profiling by Microarray

Total RNA was extracted from cells using a PureLink RNA mini kit, according to manufacturer's recommendations. One hundred and fifty nanograms of total RNA was then labeled and hybridized to a SurePrint G3 Human GE 8 × 60 K microarray chip (Agilent Technologies). All microarray experiments were conducted by the Microarray Core Facility (Stem Cell Unit, College of Medicine, King Saud University). Normalization and data analyses were performed using GeneSpring GX software (Agilent Technologies), and pathway analysis was conducted using the Single Experiment Pathway analysis feature of the GeneSpring 12.0 software package (Agilent Technologies) as described previously (66). In addition, we used the web-based software DAVID Bioinformatics Resources 6.8, where all genes that were upregulated by TGfb were uploaded into DAVID and signaling pathways were achieved. Significant changes were defined as a fold change of ≥2 and *p* < 0.02.

### 4.7. Cell Proliferation Assays

#### 4.7.1. AlamarBlue Cell Viability Assay

Cell viability was measured using alamarBlue assay reagent (AbD Serotec, Raleigh, NC, USA) according to manufacturer's recommendations. Briefly, cells were cultured in 96-well plates in 100 *μ*l of the appropriate medium before 10 *μ*l alamarBlue substrate was added at the indicated time points. Plates were then incubated in the dark at 37°C for 1 hour. AlamarBlue fluorescence was then detected using a Synergy II microplate reader (BioTek Inc.) with excitation and emission wavelengths of 530 nm and 590 nm, respectively.

### 4.8. RTCA Cell Proliferation Assay

An xCELLigence RTCA (real-time cell analysis) DP system (ACEA Biosciences Inc., San Diego, CA) was used to measure the rate of cellular proliferation according to manufacturer's protocol. Briefly, 100 *μ*l DMEM supplemented with 10% FBS was loaded onto each well of an E-plate 16 chamber slide, which was then placed inside the humidified incubator of the RTCA DP analyzer for 1 hour at 37°C to allow the membrane surface and medium to equilibrate. After 1 hour, background measurements were performed. Next, 5000 cells/100 *μ*l DMEM + 10% FBS were added per well, and measurements were recorded at 15-minute intervals for various total durations, depending on the experimental setup.

### 4.9. Transmission Electron Microscopy (TEM)

For TEM, cells were trypsinized, washed with PBS, pelleted, and then fixed in 2.5% glutaraldehyde (Cat. number 16500; Electron Microscopy Sciences) in 0.1 M phosphate buffer (pH 7.2) at 4°C for 4 hours. Next, the cells were washed in 0.1 M phosphate buffer (pH 7.2) 3 times for 30 minutes each and then treated with 1% osmium tetroxide (OsO_4_) in 0.1 M phosphate buffer (pH 7.2) for 2 hours. Cells were then dehydrated in increasing concentrations of ethanol (10%, 30%, 50%, 70%, 90%, and 100%) for 15 minutes each, before being resuspended in acetone and incubated for 15 minutes. The resulting cell suspension was then aliquoted into BEEM® embedding capsules and infiltrated firstly with a 2 : 1 acetone : resin mixture for 1 hour and secondly with a 1 : 2 acetone : resin mixture for 1 hour. Following infiltration, the BEEM capsules were centrifuged at 2500 rpm for 5 minutes and embedded in pure resin for 2 hours. The resin was then polymerized by baking in an oven at 70°C for 12 hours. Semithin sections (0.5 *μ*m thickness) were prepared and stained with 1% toluidine blue. Ultrathin sections (70 nm thickness) were prepared and mounted on copper grids and then stained firstly with uranyl acetate (saturated ethanol solution) for 30 minutes, rinsed with double distilled water and then stained with Reynold's lead citrate solution for 5 minutes before a final rinse with distilled water. The contrasted ultrathin sections were examined and photographed under a JEOL 1010 transmission electron microscope (JEOL, Tokyo, Japan).

### 4.10. Statistical Analysis

All results are presented as the mean ± SD of at least 3 independent experiments. Differences between groups were assessed using Student's *t*-test, and *p* values < 0.05 were considered statistically significant.

## Figures and Tables

**Table 1:** Osteogenesis- and adipogenesis-related genes, from the most enriched pathways, that are upregulated in TGF*β*1-treated cells.

Endochondral ossification	Actin cytoskeleton	Focal adhesion	TGF*β* signaling	MAPK signaling	Adipogenesis
VEGFA	FGF2	COL11A1	SMURF1	MAPK8	FOXO1A
ADAMTS4	FGFR1	COL3A1	MAPK8	NGFB	TRIB3
PLAT4	TMSB4X	COL4A1	SKP1	PDGFRB	PCK2
COL10A1	GNA13	COL4A2	NEDD9	RASA2	EGR2
TGFB2	PDGFA	COL4A4	ETS1	SOS2	DDIT3
PTHrP	FGF1	COL5A1	KLF11	KRAS	GADD45A
FGF2	ENAH	COL5A2	ATF3	MRAS	GADD45B
C4ST1	MSN	COL1A1	FOSB	NF1	HIF1A
FGFR1	GSN	LAMC2	SKIL	RAP1B	IRS1
PDGFRB	PDGFRB	THBS2	SMURF1	DUSP1	MEF2D
COL1	KRAS	CAV2	ZFYVE16	DDIT3	FAS
	MRAS	ARHGAP5		HSPB1	SPOCK
	SOS2	PTEN		IL1A	
		AKT3		FAS	
		PDGFA		TGFB2	
		PDGFC		MAP313	
		PGF		ZAK	
		ITGA2		AKT3	
		PDGFRB		MAP3K8	
		RAP1B		GADD45A	
		MAPK8			

**Table 2:** Genes involved in osteogenesis-related pathways that are downregulated in CYD-treated hMSCs.

Endochondral ossification	Actin cytoskeleton	Focal adhesion	TGF*β* signaling	MAPK signaling
FGF2	FGF1	ITGA6	UCHL5	STMN1
TGFB2	FGF2	STYK1	CDC2	DUSP1
PLAT	FGF5	CAV1	SMURF2	TRAF2
CALM1	FGF22	CAV2	NEDD9	BDNF
ADAMTS1	KRAS	CAV3	STAMBPL1	MAP3K5
OPG	NRAS	LAMC2	CCNB2	ACVR1C
	C11orf13	THBS1	RBL1	KRAS
	F2R	PXN	MAPK8	TGFB2
	CRK	RHOB	PARD6A	NRAS
	PAK1	PDPK1	CAV1	PPP5C
	ARHGEF7	MYLK2	TFDP1	PAK1
	VIL2	PAK1	KLF6	CRK
	PXN	MAPK8	FST	MAPK8
		BCL2	SERPINE1	
		CCND3	THBS1	
		CRK	NOG	
			HRAS	

**Table 3:** Genes involved in adipogenesis-related pathways that are upregulated in CYD-treated hMSCs.

Gene symbol	Fold change versus control
PPARGC1A	2.4170778
AGT	15.447543
GDF10	9.77814
BMP2	9.539022
UCP1	9.416412
SFRP4	6.513523
IRS4	5.973821
MEF2C	5.1696005
LPL	5.150721
PLIN1	5.144724
LEP	5.1005282
NDN	5.0982733
CNTFR	4.9715867
LIF	3.9070668
PRLR	3.855135
RXRG	3.8392398
EGR2	3.6894956
PCK1	3.6815107
CYP26A1	3.170688
TGFBI	3.1407108
STAT2	3.0656292
BMP3	2.9982927
IGF1	2.9808753
PTGIS	2.8547947
INSR	2.8151898
SLC2A4	2.747039
CEBPB	2.7001002
IRS1	2.5992496
LPIN1	2.579549
AHRR	2.5087466
IL6	2.4654725
IRS2	2.3497624
STAT5A	2.2441745
CEBPA	2.2439373
SCD	2.216643
STAT1	2.205384
SPOCK1	2.201062
SREBF1	2.0996578
EPAS1	2.0842645
MEF2D	2.0606902
BMP1	2.0168457
LPIN3	2.0103807

**Table 4:** Genes involved in both osteogenesis- and adipogenesis-related pathways that are significantly changed in TGF*β*-treated cells and CYD-treated cells.

Endochondral ossification	Actin cytoskeleton	Focal adhesion	TGFB signaling	MAPK signaling	Adipogenesis
TGFB2	FGF1	CAV2	NEDD9	TGFB2	EGR2
FGF2	FGF2	MAPK8	MAPK8	MAPK8	IRS1
PLAT	KRAS	LAMC2		KRAS	MEF2D
				DUSP1	

## Supplementary Materials

Supplementary 1 Figure S1: DAVID microarray functional gene analysis. One thousand nine hundred genes were uploaded to DAVID online software. KEGG pathway analysis showed many interested pathways which are upregulated after TGFb-1 treatment of hBMCs. Here, we show the top most upregulated pathways which include key osteoblast differentiation pathways.

Supplementary 2 Table S1: whole genome microarray mRNA data showing gene ENTITIES for TG1 versus CNT DOWN 1298 genes.

Supplementary 3 Table S2: whole genome microarray mRNA data showing gene ENTITIES for TG1 versus CNT UP 1932 genes.

Supplementary 4 Table S3: upregulated biological processes and related genes in TGFB1-treated cells using GO analysis.

Supplementary 5 Table S4: ultrastructural characteristics of CL1 cells under different treatment conditions.

Supplementary 6 Table S5: whole genome microarray mRNA data showing gene entities for CYD versus CNT up to 10,855 genes.

Supplementary 7 Table S6: whole genome microarray mRNA data showing gene entities for CYD versus CNT DOWN 2523 genes.

Supplementary 8 Table S7: real-time PCR human primer sequences used in this study.

Supplementary 9 Table S8: TAQMAN real-time PCR primers.
