# Supplementary material for: TGFβ1-Induced Differentiation of Human Bone Marrow-Derived MSCs Is Mediated by Changes to the Actin Cytoskeleton
Source: Stem Cells Int. 2018 Feb 15;2018:6913594. doi: 10.1155/2018/6913594 (PMC5832166; doi:10.1155/2018/6913594)

Supplementary Fig.1

1900 genes were uploaded to DAVID online software. KEGG pathway analysis showed many interested pathways which are upregulated after TGFb-1 treatment of hBMCs. Here we show the top most upregulated pathways which include key osteogenic pathways.

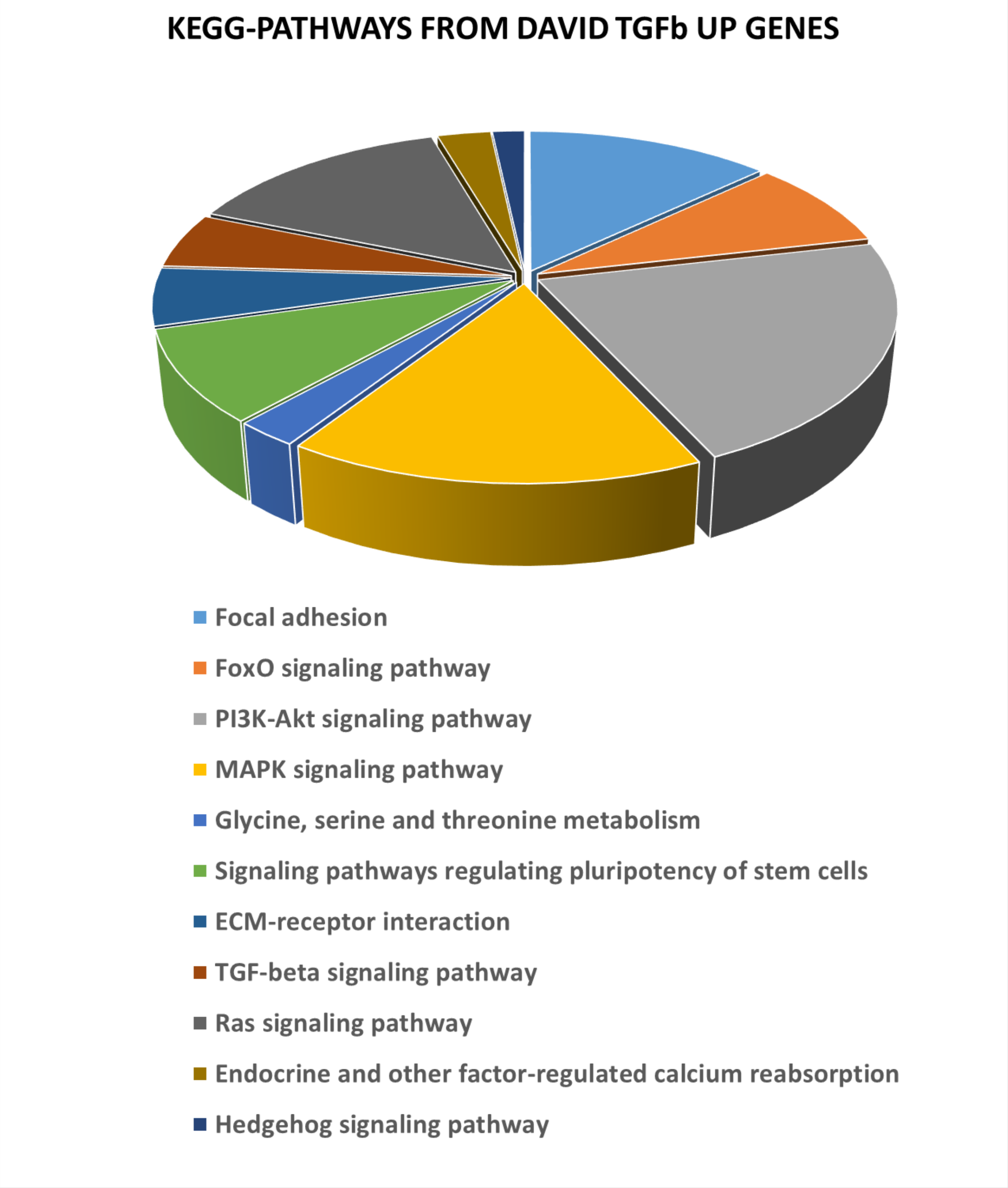

Supplement: Supplementary 1 — Figure S1: DAVID microarray functional gene analysis. One thousand nine hundred genes were uploaded to DAVID online software. KEGG pathway analysis showed many interested pathways which are upregulated after TGFb-1 treatment of hBMCs. Here, we show the top most upregulated pathways which include key osteoblast differentiation pathways. [file 6913594.f1.pdf]
