# Supplementary material for: TGFβ1-Induced Differentiation of Human Bone Marrow-Derived MSCs Is Mediated by Changes to the Actin Cytoskeleton
Source: Stem Cells Int. 2018 Feb 15;2018:6913594. doi: 10.1155/2018/6913594 (PMC5832166; doi:10.1155/2018/6913594)
Supplement: Supplementary 4 — Table S3: upregulated biological processes and related genes in TGFB1-treated cells using GO analysis. [file 6913594.f4.docx]

**Supplementary Table s3:** **Up-regulated biological processes and related genes in TGFB1 treated cells using GO analysis**

| **Extracellular Matrix** | | **Extracellular matrix Organization** | | **Protienaceous Extracellular Matrix** | |
| --- | --- | --- | --- | --- | --- |
| **Gene Symbol** | **Gene Name** | **Gene Symbol** | **Gene Name** | **Gene Symbol** | **Gene Name** |
| **MFAP2** | microfibrillar-associated protein 2 | **MFAP2** | microfibrillar-associated protein 2 | **MFAP2** | microfibrillar-associated protein 2 |
| **NDNF** | neuron-derived neurotrophic factor | **NDNF** | neuron-derived neurotrophic factor | **LOX** | lysyl oxidase |
| **LOX** | lysyl oxidase | **PDGFA** | platelet-derived growth factor alpha polypeptide | **COL7A1** | collagen, type VII, alpha 1 |
| **RARRES2** | retinoic acid receptor responder (tazarotene induced) 2 | **LOX** | lysyl oxidase | **MMRN2** | multimerin 2 |
| **COL7A1** | collagen, type VII, alpha 1 | **COL7A1** | collagen, type VII, alpha 1 | **FBLN5** | fibulin 5 |
| **MMRN2** | multimerin 2 | **FBLN5** | fibulin 5 | **MAMDC2** | MAM domain containing 2 |
| **FBLN5** | fibulin 5 | **LAMC2** | laminin, gamma 2 | **LAMC2** | laminin, gamma 2 |
| **MAMDC2** | MAM domain containing 2 | **MFAP4** | microfibrillar-associated protein 4 | **MFAP4** | microfibrillar-associated protein 4 |
| **LAMC2** | laminin, gamma 2 | **CTGF** | connective tissue growth factor | **CTGF** | connective tissue growth factor |
| **MFAP4** | microfibrillar-associated protein 4 | **DPT** | dermatopontin | **DPT** | dermatopontin |
| **CTGF** | connective tissue growth factor | **COL4A2** | collagen, type IV, alpha 2 | **COL4A2** | collagen, type IV, alpha 2 |
| **DPT** | dermatopontin | **CDH1** | cadherin 1, type 1, E-cadherin (epithelial) | **ADAMTS6** | ADAM metallopeptidase with thrombospondin type 1 motif, 6 |
| **COL4A2** | collagen, type IV, alpha 2 | **COL10A1** | collagen, type X, alpha 1 | **COL10A1** | collagen, type X, alpha 1 |
| **ADAMTS6** | ADAM metallopeptidase with thrombospondin type 1 motif, 6 | **ELN** | elastin | **ELN** | elastin |
| **COL10A1** | collagen, type X, alpha 1 | **COL5A2** | collagen, type V, alpha 2 | **COL5A2** | collagen, type V, alpha 2 |
| **ELN** | elastin | **ADAMTS4** | ADAM metallopeptidase with thrombospondin type 1 motif, 4 | **ADAMTS4** | ADAM metallopeptidase with thrombospondin type 1 motif, 4 |
| **COL5A2** | collagen, type V, alpha 2 | **COL4A1** | collagen, type IV, alpha 1 | **PODNL1** | podocan-like 1 |
| **ADAMTS4** | ADAM metallopeptidase with thrombospondin type 1 motif, 4 | **TNFRSF11B** | tumor necrosis factor receptor superfamily, member 11b | **ADAMTS12** | ADAM metallopeptidase with thrombospondin type 1 motif, 12 |
| **PODNL1** | podocan-like 1 | **HAPLN1** | hyaluronan and proteoglycan link protein 1 | **COL4A1** | collagen, type IV, alpha 1 |
| **ADAMTS12** | ADAM metallopeptidase with thrombospondin type 1 motif, 12 | **COL5A1** | collagen, type V, alpha 1 | **TNFRSF11B** | tumor necrosis factor receptor superfamily, member 11b |
| **COL4A1** | collagen, type IV, alpha 1 | **LUM** | lumican | **HAPLN1** | hyaluronan and proteoglycan link protein 1 |
| **TNFRSF11B** | tumor necrosis factor receptor superfamily, member 11b | **DMD** | dystrophin | **COL5A1** | collagen, type V, alpha 1 |
| **HAPLN1** | hyaluronan and proteoglycan link protein 1 | **ELN** | elastin | **LUM** | lumican |
| **PLAT** | plasminogen activator, tissue | **ADAMTS14** | ADAM metallopeptidase with thrombospondin type 1 motif, 14 | **VEGFA** | vascular endothelial growth factor A |
| **COL5A1** | collagen, type V, alpha 1 | **F11R** | F11 receptor | **ELN** | elastin |
| **LUM** | lumican | **TGFB2** | transforming growth factor, beta 2 | **FGF1** | fibroblast growth factor 1 (acidic) |
| **VEGFA** | vascular endothelial growth factor A | **LRP4** | low density lipoprotein receptor-related protein 4 | **ADAMTS14** | ADAM metallopeptidase with thrombospondin type 1 motif, 14 |
| **ELN** | elastin | **COL3A1** | collagen, type III, alpha 1 | **RUNX1** | runt-related transcription factor 1 |
| **FGF1** | fibroblast growth factor 1 (acidic) | **ANXA2** | annexin A2 | **SPOCK1** | sparc/osteonectin, cwcv and kazal-like domains proteoglycan (testican) 1 |
| **ADAMTS14** | ADAM metallopeptidase with thrombospondin type 1 motif, 14 | **COL6A3** | collagen, type VI, alpha 3 | **PRSS36** | protease, serine, 36 |
| **RUNX1** | runt-related transcription factor 1 | **JAM2** | junctional adhesion molecule 2 | **COL3A1** | collagen, type III, alpha 1 |
| **SPOCK1** | sparc/osteonectin, cwcv and kazal-like domains proteoglycan (testican) 1 | **COL4A4** | collagen, type IV, alpha 4 | **ANXA2** | annexin A2 |
| **TGFB2** | transforming growth factor, beta 2 | **F11R** | F11 receptor | **COL6A3** | collagen, type VI, alpha 3 |
| **PRSS36** | protease, serine, 36 | **NF1** | neurofibromin 1 | **AMTN** | amelotin |
| **COL3A1** | collagen, type III, alpha 1 | **JAM2** | junctional adhesion molecule 2 | **ADAMTS10** | ADAM metallopeptidase with thrombospondin type 1 motif, 10 |
| **ANXA2** | annexin A2 | **APBB2** | amyloid beta (A4) precursor protein-binding, family B, member 2 | **THBS2** | thrombospondin 2 |
| **COL6A3** | collagen, type VI, alpha 3 | **RECK** | reversion-inducing-cysteine-rich protein with kazal motifs | **COL4A4** | collagen, type IV, alpha 4 |
| **AMTN** | amelotin | **PXDN** | peroxidasin homolog (Drosophila) | **PXDN** | peroxidasin homolog (Drosophila) |
| **ADAMTS10** | ADAM metallopeptidase with thrombospondin type 1 motif, 10 | **NRXN1** | neurexin 1 | **NID1** | nidogen 1 |
| **THBS2** | thrombospondin 2 | **NID1** | nidogen 1 | **ADAMTS6** | ADAM metallopeptidase with thrombospondin type 1 motif, 6 |
| **COL4A4** | collagen, type IV, alpha 4 | **FOXS1** | forkhead box S1 | **COL1A1** | collagen, type I, alpha 1 |
| **PXDN** | peroxidasin homolog (Drosophila) | **COL1A1** | collagen, type I, alpha 1 | **FBN1** | fibrillin 1 |
| **NID1** | nidogen 1 | **GREM1** | gremlin 1, DAN family BMP antagonist | **THBS2** | thrombospondin 2 |
| **ADAMTS6** | ADAM metallopeptidase with thrombospondin type 1 motif, 6 | **FBN1** | fibrillin 1 | **SPARC** | secreted protein, acidic, cysteine-rich (osteonectin) |
| **COL1A1** | collagen, type I, alpha 1 | **SPARC** | secreted protein, acidic, cysteine-rich (osteonectin) | **MATN3** | matrilin 3 |
| **FBN1** | fibrillin 1 | **SERAC1** | serine active site containing 1 | **POSTN** | periostin, osteoblast specific factor |
| **THBS2** | thrombospondin 2 | **FGF2** | fibroblast growth factor 2 (basic) | **COL5A1** | collagen, type V, alpha 1 |
| **SPARC** | secreted protein, acidic, cysteine-rich (osteonectin) | **MATN3** | matrilin 3 | **MFAP5** | microfibrillar associated protein 5 |
| **SERAC1** | serine active site containing 1 | **POSTN** | periostin, osteoblast specific factor |  |  |
| **MATN3** | matrilin 3 | **COL5A1** | collagen, type V, alpha 1 |  |  |
| **POSTN** | periostin, osteoblast specific factor | **MFAP5** | microfibrillar associated protein 5 |  |  |
| **COL5A1** | collagen, type V, alpha 1 |  |  |  |  |
| **MFAP5** | microfibrillar associated protein 5 |  |  |  |  |
