# Supplementary material for: TGFβ1-Induced Differentiation of Human Bone Marrow-Derived MSCs Is Mediated by Changes to the Actin Cytoskeleton
Source: Stem Cells Int. 2018 Feb 15;2018:6913594. doi: 10.1155/2018/6913594 (PMC5832166; doi:10.1155/2018/6913594)
Supplement: Supplementary 5 — Table S4: ultrastructural characteristics of CL1 cells under different treatment conditions. [file 6913594.f5.docx]

**Supplementary Table s4: Ultrastructural characteristics of CL1 cells under different treatment conditions**

|  | | **CNT** | **+SB** | **+TGFB** | **+CYD** | **TGFB+CYD** |
| --- | --- | --- | --- | --- | --- | --- |
| **ACTIN** | **Prominence** | +++ * | + | ++++ | + | +++ |
|  | **Form** | Aggregated in bundles | Scattered | More Bundles Forms | Scarce | Aggregated |
|  | **Location** | Peri-nuclear/ throughout cytoplasm | Throughout cytoplasm | Peri-nuclear/at one cell pole | Throughout cytoplasm | Throughout cytoplasm |
| **Rough Endoplasmic Reticulum (rER)** | **Prominence** | ++ | ++ | +++ | ++ | ++++ |
|  | **Morphology** | Long stalks | Short stalks | Long stalks | Long stalks | Cystically dilated |
| **Mitochondria** | **Maturity** | Most are mature | Most are Premature | Most are mature | Mature and premature | Mature and premature |
|  | **Size** | Medium to large | Most are small | Small to medium | Small to medium | Small, Medium, and Large |
| **Nucleus** | **Prominence of Nucleoli** | +++ | ++ | ++ | +++ | ++ |
|  | **Distribution of heterochromatin** | ++ | + | + | + | + |
|  | **Infoldings of nuclear membranes** | ++ | + | ++ | ++ | + |
| **Lysosomal Structures** | **Primary** | + | + | + | + | + |
|  | **Secondary** | +++ | ++ | ++ | + | +++ |
| **Surface Structures** | **Microvilli** | +++ | + | ++++ | ++ | + |
|  | **Processes** | + | + | + | + | + |
|  | **Blebs** | + | ++ | + | ++ | ++ |
| **Myelin Figures** | **Prominence** | +++ | + | + | + | +++ |

- +, slight; ++, mild; +++, moderate; ++++, prominent
