# Supplementary material for: TGFβ1-Induced Differentiation of Human Bone Marrow-Derived MSCs Is Mediated by Changes to the Actin Cytoskeleton
Source: Stem Cells Int. 2018 Feb 15;2018:6913594. doi: 10.1155/2018/6913594 (PMC5832166; doi:10.1155/2018/6913594)
Supplement: Supplementary 8 — Table S7: real-time PCR human primer sequences used in this study. [file 6913594.f8.docx]

Supplementary Table s7: Real-time PCR human primer sequences used in this study

| **Gene Name** | **Forward Primer (5´–3´)** | **Reverse Primer (5´–3´)** |
| --- | --- | --- |
| ***GAPDH*** | CTGGTAAAGTGGATATTGTTGCCAT | TGGAATCATATTGGAACATGTAAACC |
| ***Runx2*** | CACCATGTCAGCAAAACTTCTT | ACCTTTGCTGGACTCTGCAC |
| ***ALPL*** | GACGGACCCTCGCCAGTGCT | AATCGACGTGGGTGGGAGGGG |
| ***OCN*** | GGCAGCGAGGTAGTGAAGAG | CTCACACACCTCCCTCCTG |
| ***PPARG*** | TTCTCCTATTGACCCAGAAAGC | CTCCACTTTGATTGCACTTTGG |
| ***AP2*** | GCCAGGAATTTGACGAAG TC | TGGTTGATTTTCCATCCCAT |
| ***LPL*** | CTTGGAGATGTGGACCAGC | GTGCCATACAGAGAAATCTC |
| ***ADIPOQ*** | GCAGTCTGTGGTTCTGATTCCATAC | GCCCTTGAGTCGTGGTTTCC |
