# Supplementary material for: TGFβ1-Induced Differentiation of Human Bone Marrow-Derived MSCs Is Mediated by Changes to the Actin Cytoskeleton
Source: Stem Cells Int. 2018 Feb 15;2018:6913594. doi: 10.1155/2018/6913594 (PMC5832166; doi:10.1155/2018/6913594)
Supplement: Supplementary 9 — Table S8: TAQMAN real-time PCR primers. [file 6913594.f9.docx]

Supplementary Table s8: TAQMAN Real-time PCR primers

| Gene ID | Assay ID | Cat no. |
| --- | --- | --- |
| GAPDH | Hs02758991_g1 | 4331182 |
| TAGLN | Hs01038777_g1 | 4331182 |
| TPM1 | Hs00165966_m1 | 4331182 |
| ACTA2 | Hs00426835**_g1** | 4331182 |
| CDH11 | Hs00901475_m1 | 4331182 |
| COL10A1 |  |  |
| B3GLNT2 |  |  |
| POSTN | Hs00170815_m1 | 4331182 |
| CNN1 | Hs00154543_m1 | 4331182 |
| FBLN5 |  |  |
| MUC15 |  |  |
